# Analysis of Maize (*Zea mays* L.) Seedling Roots with the High-Throughput Image Analysis Tool *ARIA* (Automatic Root Image Analysis)

**DOI:** 10.1371/journal.pone.0108255

**Published:** 2014-09-24

**Authors:** Jordon Pace, Nigel Lee, Hsiang Sing Naik, Baskar Ganapathysubramanian, Thomas Lübberstedt

**Affiliations:** 1 Department of Agronomy, Iowa State University, Ames, Iowa, United States of America; 2 Department of Mechanical Engineering, Iowa State University, Ames, Iowa, United States of America; Mahatma Phule Agricultural University, India

## Abstract

The maize root system is crucial for plant establishment as well as water and nutrient uptake. There is substantial genetic and phenotypic variation for root architecture, which gives opportunity for selection. Root traits, however, have not been used as selection criterion mainly due to the difficulty in measuring them, as well as their quantitative mode of inheritance. Seedling root traits offer an opportunity to study multiple individuals and to enable repeated measurements per year as compared to adult root phenotyping. We developed a new software framework to capture various traits from a single image of seedling roots. This framework is based on the mathematical notion of converting images of roots into an equivalent graph. This allows automated querying of multiple traits simply as graph operations. This framework is furthermore extendable to 3D tomography image data. In order to evaluate this tool, a subset of the 384 inbred lines from the Ames panel, for which extensive genotype by sequencing data are available, was investigated. A genome wide association study was applied to this panel for two traits, *Total Root Length* and *Total Surface Area,* captured from seedling root images from WinRhizo Pro 9.0 and the current framework (called *ARIA*) for comparison using 135,311 single nucleotide polymorphism markers. The trait *Total Root Length* was found to have significant SNPs in similar regions of the genome when analyzed by both programs. This high-throughput trait capture software system allows for large phenotyping experiments and can help to establish relationships between developmental stages between seedling and adult traits in the future.

## Introduction

The maize (*Zea mays* L.) root is designed to provide anchorage as well as to secure uptake of water and nutrients, including nitrogen (N), in an efficient manner [1], [2]. Maize roots are formed partly during embryonic and partly during post-embryonic development [3]. There are five main types of roots in maize: crown, seminal, primary, lateral, and brace roots [4]. The major portion of root biomass of mature plants is derived from postembryonic, shoot-borne roots. These postembryonic roots include crown roots, formed below soil surface, and brace roots, formed above soil surface [5]. Their function is important to plant performance as they are responsible for the majority of water and nutrient uptake in maize [5].

Two to three week old seedling root systems are made up of primary roots, lateral roots, seminal roots, and root hairs [4], [6]. Lateral roots branch outward from the primary root. These root types are called the axial roots and determine root architecture. Lateral roots increase the surface area of the root system and all root types contribute to water and nutrient uptake [2], [7], [8]. Moreover, lateral roots contain root initiation points, leading to secondary, tertiary, and higher order root structures, with major influence on the overall root architecture of the root stock [2].

There is extensive genetic variation in root architecture. However, root traits have not been considered by plant breeders to select for improved nutrient uptake efficiency or yield improvement due to the difficulty in measuring root traits and their quantitative mode of inheritance [9]. Studying adult roots using maize “shovelomics”, a high-throughput phenotyping technique that measures adult root traits, is time consuming and laborious. This method of phenotyping is also destructive because roots are dug out of the ground. This limits the number of experiments that can be completed in a season [10]. Changes in maize root architecture may strongly affect yield [11]. Seminal roots play a key role in the acquisition of immobile and mobile nutrients such as phosphorus and nitrogen respectively and can determine spatial and temporal domains of its environment and inter-root competition [6]. The relationship between seminal root biomass in hydroponics and root lodging in a field study focusing on root strength and pulling resistance has been explored. Respective correlations were low, but statistically significant. Correlations found in hydroponic seedling root traits compared to adult field traits were r = 0.44* for shoot weight and adult plant height, and r = 0.22* for lateral root length with brace root development [12], [13].

Seedling phenotyping takes less time, is less laborious, and can be repeated many times during the year allowing for quicker turnover of results. Positive but low correlations were found between maize seedling and adult root traits, such as number of seminal roots and weight of seminal roots to root pulling resistance (r = 0.07 and r = 0.36*, respectively) [14]. Expanding the number of seedling root traits and improving respective phenotyping procedures, may increase the chance of capturing strong relationships between different growth stages in maize.

Using digital imaging software to automate phenotypic analysis is an innovative and efficient way of accurately taking measurements of plant physiological traits [15]–[19]. Roots have been difficult to phenotype in a high throughput manner due to a lack of simple access and their highly plastic nature. With the development of custom root analysis systems, quantitative studies of root systems are now possible [20], [21]. There are several software frameworks that extract root morphology traits in two-dimensions in various hierarchies of automation. This ranges from manual root labeling like DART (Le Bot and Serra, 2009), to semi-automated software like WinRhizo (Pro, 2004), a commercial root analysis tool, and EzRhizo [22], a freely available software, all the way to full integrated imaging-analysis platforms like SmartRoot [23] for small root systems and recent platforms, allowing for automated measurements as well as invoking a ranking system for root traits [17].

These software frameworks have substantially enhanced the research community’s ability to efficiently analyze and accumulate massive amounts of data. They also pioneered the utilization of graphical user interface (GUI) that enables ease of use. However, most of these software frameworks are either expensive, not expandable to increased (or decreased) dimensions, or cannot be fine-tuned to a specific setup. We developed an open-source, modular, easy-to-use and efficient root system architecture characterization software called *ARIA* (Automatic Root Image Analysis). This is based on a mathematically rigorous approach of converting root images into graphs. We show how extracting a variety of traits becomes a simple process of utilizing various graph algorithms. There are several major advantages to such a graph based approach to extracting root system traits: (a) graph based methods are well-studied and have very fast and efficient algorithms (for example, used in Google, Facebook, most GPS devices etc.) that enable fast, real time data analysis, (b) graph based methods are easily scalable (having almost linear computational complexity) and, hence, can be easily extended to larger problem sizes without compromising on time (with direct implication to large 3D tomography datasets), and (c) a graph-based approach is generic. That is, by making trivial modifications to the definitions of parameters like edges, weights, and labels, a huge variety of traits can be accessed. This makes a graph based framework trivially extendable. Furthermore, graphs are dimension independent, and hence this framework is trivially extendable to 3D root image analysis.

In this study, the utility of *ARIA* has been tested by phenotyping 384 maize inbred lines using scanned images of seedling roots. These data were then applied to a genome wide association study (GWAS) to detect marker-trait associations. Measurements of the trait Total Root Length were analyzed for a comparative GWAS study, as this is the only trait shared between the current platform WinRhizo Pro 9.0 and *ARIA*. The objective of this study is to show that our new and freely accessible root phenotyping software *ARIA* is a fast and accurate platform for automated phenotyping, with the potential of adding additional features when compared to the established software WhinRizo Pro 9.0. For both programs, significant marker trait associations were found using a general linear model. Also, phenotypic measurements with both programs were compared using a 74 maize inbred line panel [24] to further validate utility of *ARIA*. The results of this study show that *ARIA* is an accurate and dependable tool for completing large phenotyping experiments, needed for many quantitative genetic studies. Its flexibility makes *ARIA* a very useful tool to breeders and biologists studying root architecture.

## Results

### Root Traits Captured by ARIA

**Figure 1:**
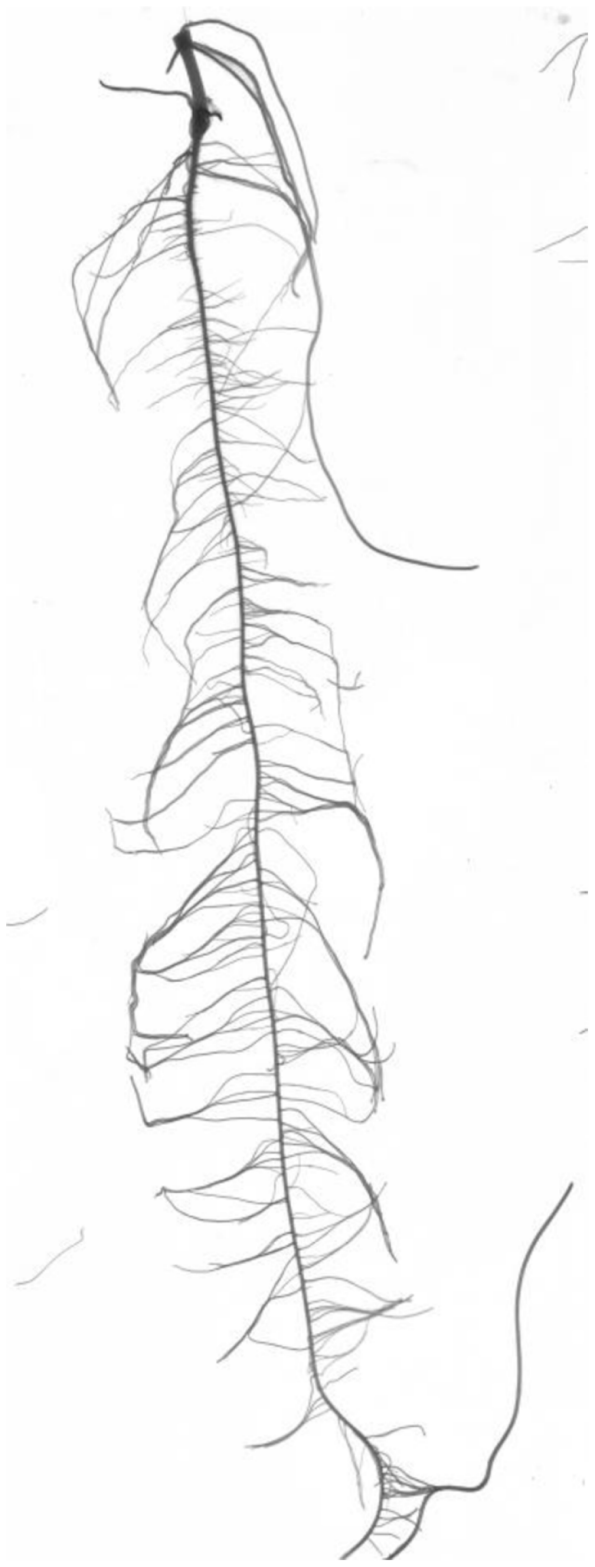
Image of a 14 day root.

Using *ARIA*, 27 different root traits were extracted from each scanned image of seedling roots (Table 1). Some traits are more suitable for 3D root scan image analysis such as Depth, Width, and the Width/Depth ratio. All simple statistics as well as heritability estimates for all root traits are found in Table S2. This program is free software and can be accessed using the following link: http://www3.me.iastate.edu/bglab/pages/software.html *ARIA* captures more traits than existing programs such as WinRhizo Pro 9.0, which lists eight different traits that can be obtained from a single root scanned image when buying a standard package. *ARIA* is fully automated with the ability to capture up to three separate seedling roots from a single image, and to conduct all analyses with limited user interference. Each image was a high resolution scan (around 4400×6200 pixels) of three seedling roots placed side-by-side (Figure 1). Within each image the bounding boxes were automatically identified for each root. Each of the three roots is then individually analyzed and its 27 traits extracted. Data is then exported into an Excel file. This process takes approximately 20 seconds on a standard desktop (2.8 GHz machine). We used a total of 1059 images, each containing up to three roots per image. *ARIA* ran autonomously and extracted traits within 12 hours, allowing for fast turnaround of phenotypic data. Thus, trait capture is very fast and efficient when analyzing multiple roots of large experiments.

**Table 1:** Traits captured by *ARIA.*

Trait Name	Symbol	Trait Description
**Total Root Length**	TRL	Cumulative length of all the roots in centimeters
**Primary Root Length**	PRL	Length of the Primary root in centimeters
**Secondary Root Length**	SEL	Cumulative length of all secondary roots in centimeters
**Center of Mass**	COM	Center of gravity of the root.
**Center of Point**	COP	Absolute center of the root regardless of root length.
**Center of Mass (Top)**	CMT	Center of gravity of the top 1/3 of the root (Top).
**Center of Mass (Mid)**	CMM	Center of gravity of the middle 1/3 root (Middle).
**Center of Mass (Bottom)**	CMB	Center of gravity of the bottom 1/3 root (Bottom).
**Center of Point (Top)**	CPT	Absolute center of the root regardless of root length (Top).
**Center of Point (Mid)**	CPM	Absolute center of the root regardless of root length (Middle).
**Center of Point (Bottom)**	CPB	Absolute center of the root regardless of root length (Bottom).
**Maximum Number of Roots**	MNR	The 84th percentile value of the sum of every row.
**Perimeter**	PER	Total number of network pixels connected to a background pixel.
**Depth**	DEP	The maximum vertical distance reached by the root system.
**Width**	WID	The maximum horizontal width of the whole RSA.
**Width/Depth ratio**	WDR	The ratio of the maximum width to depth.
**Median**	MED	The median number of roots at all Y-location.
**Total Number of Roots**	TNR	Total number of roots.
**Convex Area**	CVA	The area of the convex hull that encloses the entire root image
**Network Area**	NWA	The number of pixels that are connected in the skeletonized image
**Solidity**	SOL	The fraction equal to the network area divided by the convex area
**Bushiness**	BSH	The ratio of the maximum to the median number of roots.
**Length Distribution**	LED	The ratio of TRL in the upper one-third of the root to the TRL.
**Diameter**	DIA	Diameter of the primary root.
**Volume**	VOL	Volume of the primary root
**Surface Area**	SUA	Surface area of the primary root.
**SRL**	SRL	Total root length divided by root system volume

### Seedling Trait Correlations

**Figure 2:**
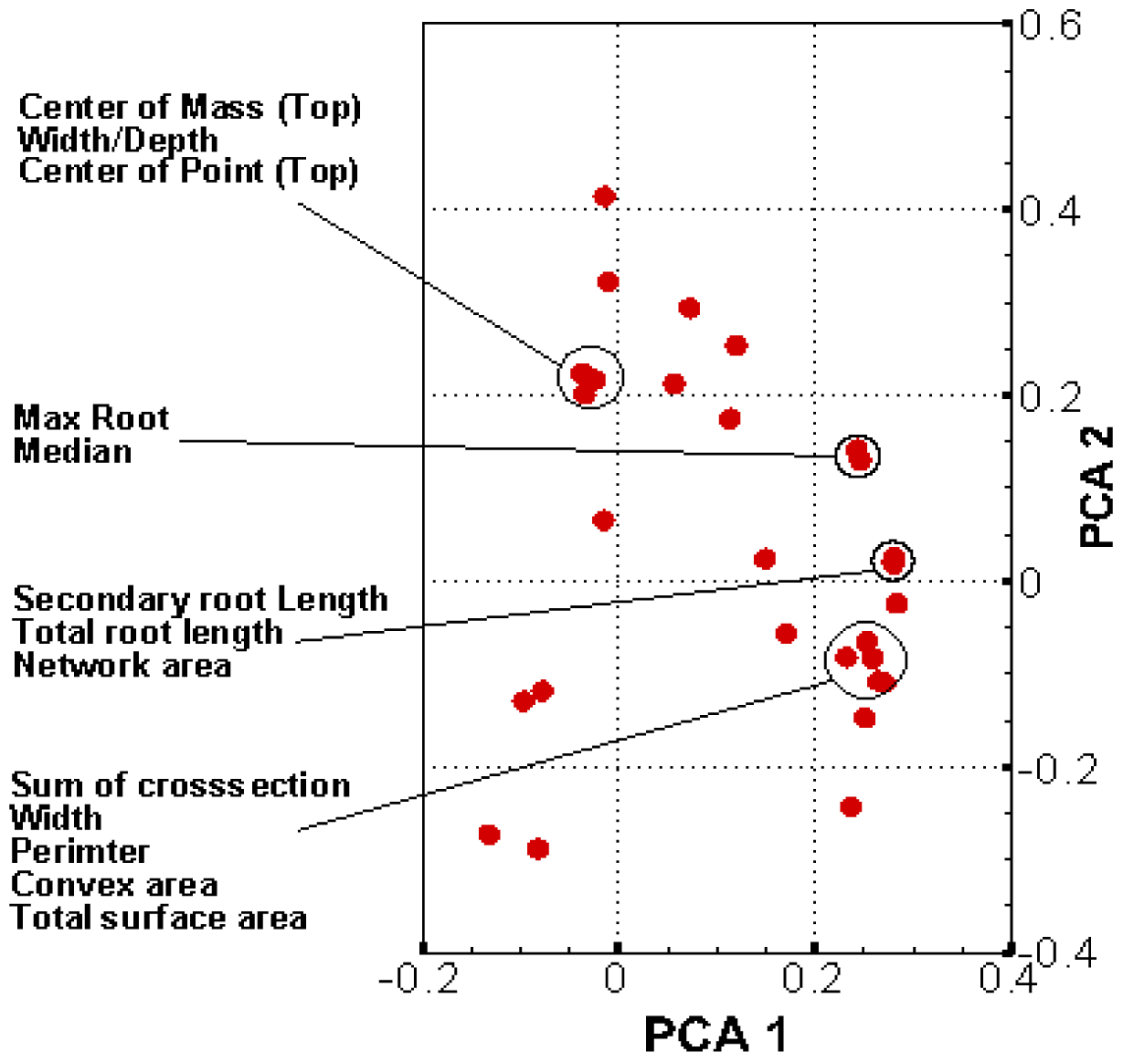
PCA plot of all *ARIA* trait Pearson correlations, clusters of traits have been marked showing which traits are closest related.

Pearson correlations were calculated using SAS 9.3 for all 27 seedling root traits compared to one another. Correlations between traits (Table S1) ranged from very close (r = 0.998) between traits such as secondary root length and PRL to no significant correlations (r = −0.061) for TRL and BSH. BSH did not correlate closely with other root traits with the highest r^2^ value of 0.166. Similarly, SRL did not show close correlations with other seedling root traits, with its closest correlation of 0.5 with TSA. Conversely, it was found that seedling root trait DEP had close correlations with various other root traits, especially with PRL (r = 0.95). A principle component analysis (PCA) was conducted to visualize trait relationships. The first two components explain 45.9% of the variation (with PCA 1 explaining 35.5%). Based on the first two principle components (Figure 2), there are four trait clusters. These clusters are comprised of (1) CMT, WDR, CPT, (2) MNR, and MED, (3) SEL, TRL, NWA, and (4) SCS, WID, PER, CVA, and TSA. All of these traits had close correlations within clusters while traits outside of clusters were not closely correlated (Table S1).

### Validation of Measurements

In order to validate measurements made by *ARIA* with those obtained by WinRhizo Pro 9.0 (Regent Instruments, Quebec, Canada), the same images of hydroponically grown maize seedling roots were analyzed by both programs and data compared. *Total Root Length* was found to be closely correlated with r = 0.97 (P = 0.0001) when analyzing data within the Ames Panel. For the ASI panel, total root length was correlated between the two programs at r = 0.92 (P = 0.0001), and root surface area was closely correlated at r = 0.90 (P = 0.0001). Broad sense heritabilities (H^2^) were calculated for both association mapping populations (Table 2). Heritability estimates were generally higher for measurements extracted using *ARIA* at H^2^ = 0.42 compared to H^2^ = 0.41 for total root length measured in the Ames Panel, as well as root surface area in the ASI panel with H^2^ = 0.54 using *ARIA* compared to H^2^ = 0.50 using Whin Rhizo Pro 9.0.

**Table 2:** Comparison of repeatability estimates for both WhinRhizo Pro 9.0 and *ARIA.*

Analyzing tool	Trait	Heritability (H^2^)
**WinRhizo Pro 2009**	Total Root Length (Ames Panel)	.41
***ARIA***	Total Root Length (Ames Panel)	.42
**WinRhizo Pro 2009**	Total Root Length (ASI Panel)	.42
***ARIA***	Total Root Length (ASI Panel)	.42
**WinRhizo Pro 2009**	Root Surface area (ASI Panel)	.50
***ARIA***	Root Surface area (ASI Panel)	.54

### Genome Wide Association Study Using ARIA vs WinRhizo

**Figure 3:**
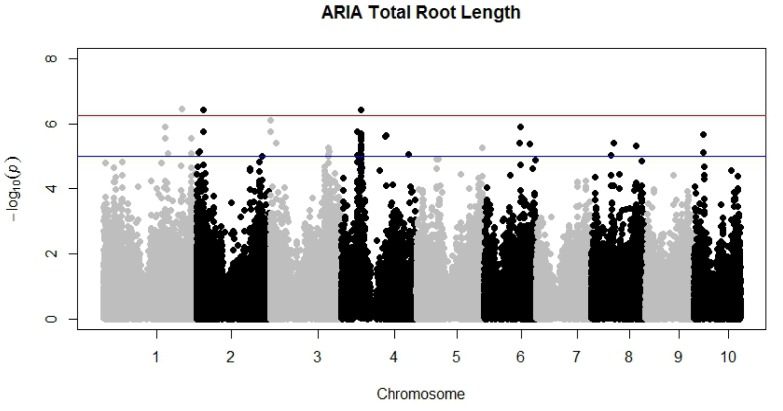
Manhattan plot displaying all 10 maize chromosomes, showing SNP markers significantly associated with trait Total Root Length measured with *ARIA*. Significant SNPs are located on chromosomes 1, 2, and 4.

**Figure 4:**
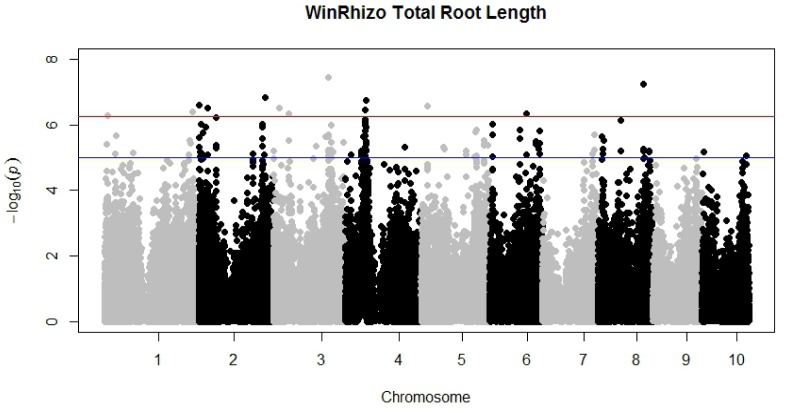
Manhattan plot displaying all 10 maize chromosomes, showing SNP markers significantly associated with trait Total Root Length measured with WinRhizo, significant SNPs are consistent with *ARIA*, with additional SNPs on chromosomes 3, 5, 6, and 8.

A GWAS experiment was conducted in order to show the utility of this new program and its ability to analyze many root images in a high-throughput manner compared to WinRhizo, the current platform used. Further GWAS analyses will be documented in a future publication. TRL was extracted from a single scan of three roots from each inbred line. This process was repeated three times, once for each replication. Analysis of TRL measured with both *ARIA* and WinRhizo combined with genotypic information on 135,311 single nucleotide polymorphism markers across the entire genome identified significant associations at p<5.3×10^−7^. Markers found to be significant were located on chromosomes 1, 2, and 4 for *ARIA* (Figure 3) while WinRhizo analysis resulted in additional SNPs on chromosomes 3, 5, 6, and 8 (Figure 4). Both programs identified significant markers in similar regions of the genome specifically on chromosome 2 and chromosome 4. Moreover, significant SNPs on Chromosome 4 were identical for both programs.

## Discussion

### Quality of ARIA Trait Estimates, Limitations and Prospects

*ARIA* is a reliable program that results in accurate measurements comparable to established programs such as WinRhizo Pro 9.0. The close correlation and higher heritability estimates of TRL and TSA are encouraging for using *ARIA* to obtain accurate measurements in future quantitative studies. A limitation for using *ARIA* in the current study was that only three roots were analyzed at a time. *ARIA* can be extended to allow a larger number of roots to be analyzed within a single image, depending on the scanning or image capture device. Since *ARIA* can automatically crop pictures for the user, keeping roots separate is important for accurate measurements, as crossed over roots could cause uneven cropping or erroneous paths. When comparing the amount of time needed to extract root measurements with *ARIA* and extracting measurements with WinRhizo, *ARIA* simplifies the process and cuts the time taken measurements to less than half the amount of time needed for WhinRhizo. This is in part due to the automatic cropping system as well as exporting measurement values into an Excel spreadsheet all at once within 20 seconds per seedling root. In WhinRhizo, each root has to be cropped manually; data are extracted into a.txt file, which needs to be edited for data analysis. Exporting data directly into a user friendly format *ARIA* by-passes all of these intermediate steps. The current version of *ARIA* is automated for roots exhibiting a distinguishable primary root. However, *ARIA* should work equally well with multiple equal order roots with minor changes due to the graph based formulation: *ARIA* finds all lengths of roots as distances from kernel to root tips and subsequently picks the single longest root (this can be modified to account for multiple equal order roots, for example in rice). A potential limitation is when a secondary root curl ends exactly at the primary root. This creates circular loops in the graph that impair further analysis. However, none of the 3000 images analyzed exhibited this issue. A way to resolve this minor issue is to consider a quasi-temporal approach to ‘growing’ the graph vertex-by-vertex that will distinguish these overlaps (work in progress).

The graph based formulation makes this framework easily extendable to multiple purposes [25], [26]. This same framework of trait extraction has been applied in other disciplines including chemistry [25] and materials science [26]. Examples of extensions include 3D phenotyping where magnetic resonance imaging (MRI), X-ray or optical tomography data can be curated and traits extracted. Furthermore, *ARIA* can work with a variety of data formats including photographs, scanned images, microscopy images as well as X-ray based reconstructions.

### Significance of ARIA

While current root analysis programs are available to make measurements of root traits, none currently offers the flexibility and functionality as *ARIA*. When comparing WinRhizo 9.0 to *ARIA*, the larger numbers of traits that can be captured, ability to capture 3D image measurements, and shorter time spent to extract trait measurements from images, are key advantages of. *ARIA*, automatically crops root images, after a mouse click defines the starting point for measurements. Furthermore, *ARIA* has the ability to mark a batch of images enabling batch analysis. In *ARIA*, measurements are exported into an Excel spreadsheet, while WinRhizo gives a text file that must be converted. *ARIA’s* ability to do this automatically makes this program high-throughput and decreases chances of human error. Another key advantage to this program is the fact that measurement capabilities can easily be added, as additional key architectural attributes of roots are determined.

**Figure 5:**
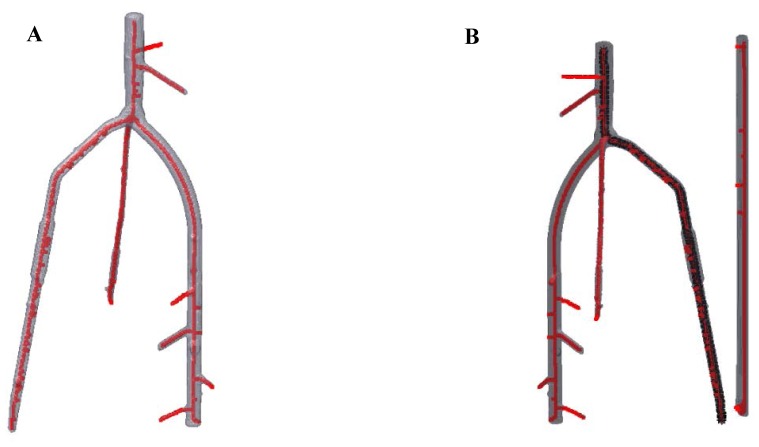
Demonstration of a 3D image analysis. A) Skeletonization of a 3D root image using *ARIA*. B) After skeletonization process is complete, the primary root is found and highlighted in black for accurate measurement of PRL measured at 1479 pixels, TRL measured at 582 pixels, and SEL with a measurement of 897 pixels. Pixel count can be converted to standard measurement notation with the inclusion of a baseline ruler to count pixels per cm or inch.

**Figure 6:**
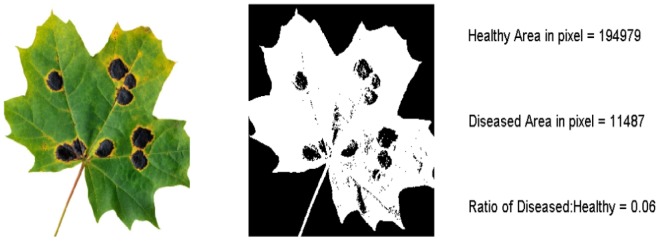
Analysis of a diseased maple leaf, *ARIA’s* flexible mainframe will allow multiple uses of the program beyond root phenotyping.

Using *ARIA*, mapping studies for root traits can be implemented on a larger scale due to the reduced time needed for phenotyping. This software system aids plant scientists by relieving the phenotyping bottleneck for quantitative traits such as root architectural traits by adding to existing technologies in phenomics [27]. Not only is this program fast, its ability to analyze both 2D and 3D images also offers a unique opportunity to look at the same traits, with the same analysis program, but from two different perspectives. Previous programs such as RootReader2D [28] and RootReader3D [29] offer extensive trait collection, but are hindered by the fact that each program is restricted to analyze at either 2D or 3D. *ARIA* in comparison is able to not only analyze 2D flat plane images such as those presented here, but also 3D images of roots. To show this feature, a simple 3D image of a root was analyzed using *ARIA* (Figure 5). Here, we demonstrate that skeletonization and outlining of the primary root can be completed as in 2D. The actual measurements of select traits have also been included in pixels (Figure 5). Based on multiple points of view of the same root system, *ARIA* extracts 27 root traits in a single root analysis. Figure 6 shows how the mathematical foundation (graph based analysis) coupled with the open-source framework can be trivially extended to other trait extraction.

A similar program described by Pascuzzi [17] was used to analyze rice varieties within a gel medium. This program has the ability to capture many of the same traits as *ARIA*. The major advantage of *ARIA* is that it can directly analyze those same gel medium images in both 2D and 3D formats. This adds to the flexibility of this free access program. Existing phenotyping systems can utilize this analysis tool without changing their growth procedures, whereas the other program is not as dimensionally flexible. No changes need to be made in the GUI or procedures to analyze images. This allows for an expanded number of environmental conditions, whether controlled by humans or nature, in which root architecture could be studied and for connections between how root develop in a hydroponic environment compared to soil or other growth medium.

### Exploring Roots as a Model for Selection

Large scale mapping studies such as quantitative trait locus (QTL) mapping and GWAS require large mapping populations that must be phenotyped in an accurate manner. Genomic selection (GS) [30] is a method in which a training population is used to collect phenotype information and coupled with extensive genetic information. Then, a model is developed to make predictions for the performance of traits of interest, solely based on genetic information. This requires massive amounts of phenotypic information that are highly accurate, especially for plastic traits such as root architecture [31]. Currently, root architecture is not used for selection, because of the resources needed for extensive phenotyping and the quantitative mode of inheritance of root traits [9]. New phenotyping software such as *ARIA* may facilitate to include root architecture in selection schemes. Comparative GWAS for TRL obtained with both WinRhizo Pro 9.0 and *ARIA* identified similar or identical regions of the genome associated with this trait. Associations found in only one program may be due to low power of detecting a polymorphism with small genetic effect.

The major goal was to develop an easy to use image software analysis tool for measuring root traits from simple scans or photographs. A free to use software platform with ability to investigate both 2D and 3D root architectural characteristics for plants has been developed to facilitate measuring multiple root traits in a high-throughput, accurate manner. We compared this new program to existing programs. *ARIA* showed close correlations to traits measured with established software, supporting accurate measurements. The 27 root traits measured give an example for the utility of this program and offer an extensive amount of traits to be studied for large scale phenotypic analysis of roots or mapping studies looking at the genetic control of root architecture. Future studies using this program include root characterization for particular maize or other plant species of interest as well as phenotyping for quantitative trait studies such as GWAS, QTL mapping, and GS.

## Materials and Methods

### ARIA (Automatic Root Image Analysis)

*ARIA* is custom software written in the programming framework, MATLAB (Natick, Massachusetts, United States). *ARIA* has a user friendly GUI interface to enable easy and rapid data extraction. The operational concept of the software is to convert the root image (after standard image pre-processing) into a graph. The software framework can read in most standard image formats. Each image is loaded (Figure 1), and after a sequence of pre-processing steps, converted into a graph. A graph is a mathematical construct consisting of a set of vertices that are connected by a set of edges. This is done by labeling each pixel of the root image into a vertex, and linking nearest neighbor pixels with edges. The key steps of the software are:

#### a) Thresholding

**Figure 7:**
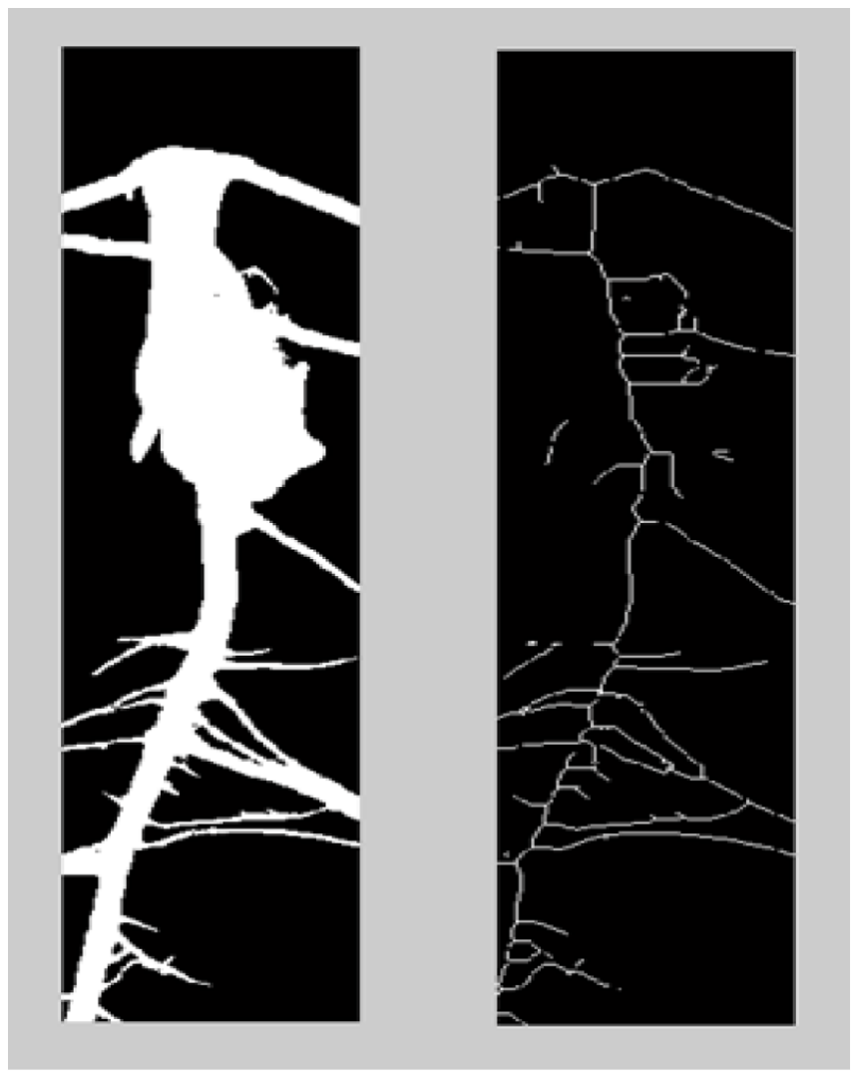
Thresholding and skeletonization stages.

The background is first identified (using morphological operations in Matlab) and renormalized to black. This effectively eliminates most of the background signal. Then the image threshold is calculated using Otsu’s method. The grey scale image is converted into a black and white image. This is done by comparing the intensity of each pixel with a threshold value. The pixel is marked as black (or white), if it’s grey scale value is smaller (or larger) than the threshold (Figure 7).

#### b) Connected components

Since the root is one large connected system, everything else that is not connected to the root can be removed from the image. This idea is encoded in the graph concept of connected components, which enumerates all the distinct connected components in the image. The largest connected component is the root, all the other connect components are noise or other foreign artifacts. Note that if the image resolves finer root hairs (which our imaging process does not do) these will still be part of the largest connected component.

#### c) Skeletonization

A ‘wire-frame’ skeleton of the binary image is constructed by thinning (or eroding). Skeletonization is a fundamental tool with many applications in image processing and visualization. Here, skeletonization is essential to identify and distinguish between the primary and secondary roots (Figure 7).

#### d) Primary and secondary root identification

**Figure 8:**
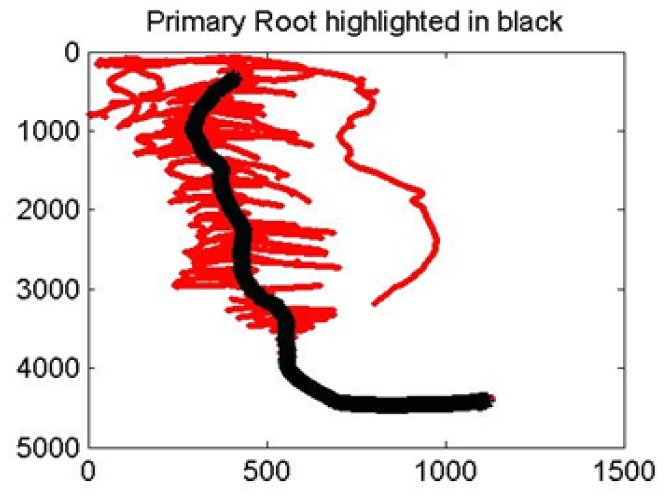
Automated identification of primary and secondary roots.

The primary root is identified as the graph path that has the longest path length (Figure 8). This is accomplished by Dijkstra’s algorithm to estimate shortest paths between two points of the graph [32]. Dijkstra’s algorithm is used to compute the shortest paths from each free end of the root to every other free end. The longest “shortest path” is identified as the primary root. Secondary roots are identified easily by subtracting the primary root from the original image and enumerating the remaining distinct connected components.

#### e) Graph querying and post processing

**Figure 9:**
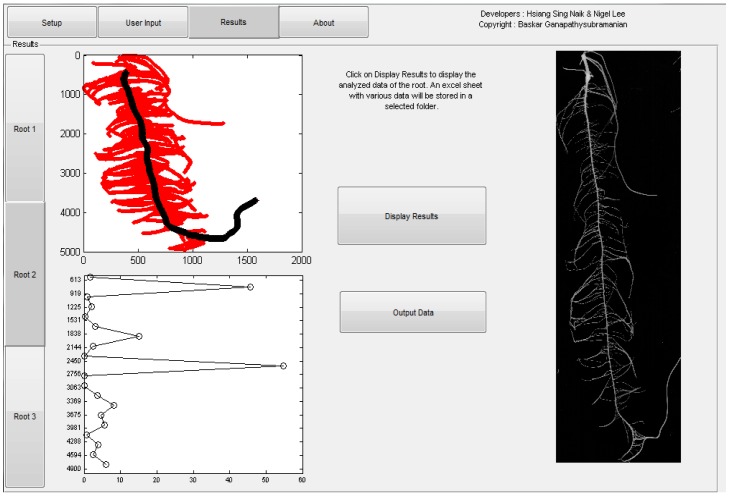
Screen capture of the *ARIA* framework. The picture on the right is the root image. The plot on the top left is automated identification of primary and secondary roots. The graph on the bottom left represents detailed analysis of root architecture, specifically a histogram of secondary roots across each 10% of the primary root.

The graph is queried to construct several traits starting from simple traits like total root length, to more complex measures like bushiness. All data are exported into an Excel sheet for ease of analysis and use. This will allow one to place a series of images for analysis at a time and export it to Excel. The data are also displayed on the GUI. All traits are analyzed automatically and can be viewed when clicking display results (Figure 9).

#### Plant materials

The first association mapping population or “Ames panel” is comprised of 384 inbred lines obtained from the USDA-ARS North Central Regional Plant Introduction Station (NCRPIS) in Ames, Iowa. All lines used in this study are a subset of a larger collection of lines called the Ames panel [33], consisting of 2815 maize inbred lines conserved at USDA-ARS NCRPIS. The 384 lines were selected based on maturity in view of future field trials in central Iowa. The second panel of 74 maize inbred lines called “ASI panel” includes ex-PVPs (Plant Variety Protection) and Germplasm enhancement of Maize (GEM) inbred lines [24].

### Root Phenotyping

#### Cigar Roll Growth Conditions

A paper roll assay described by [24] was used for germination and growth of maize seedlings. Seedlings were grown in 2 L glass beakers filled with 1.4 L of sterilized water. Seedlings were placed in a growth chamber for 14 days at 16/8 hrs light/darkness (25/22°C). Light intensity was 200 µmol photons m^−2^s^−1^, and a relative humidity maintained at 65%. Each paper roll with four seedlings was considered as experimental unit. After 14 days seedlings were removed from the growth chamber and phenotypic traits measured. If not all traits were measured the same day, plants were preserved in 30% ethanol to prevent aging of roots.

#### Image Acquisition

Seedling roots were imaged using a high resolution scanner. Three separate seedling roots were imaged at a time using an EPSON Expression 10000 XL scanner system (Copyright © 2000–2014 Epson America, Inc).

### Phenotype Data Analysis

#### Experimental Design

Ames panel lines were grown in three experiments starting June 12, 2012, July 3, 2012, and October 5, 2012. Each experiment was grown in the same growth chamber and at the same growing conditions, as described above. Lines were grown in a completely randomized design (CRD) and trait data were collected per experimental unit: three seedlings out of four within each seed roll were sampled, to eliminate possible outliers within lines, and means taken. The ASI panel of 74 maize inbred lines were grown under the same conditions and replicated twice under one experiment. Analysis of variance of root traits was performed, the additive model for analysis of variance was:
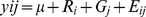
where *y_ij_* represents the observation from the *ijth* experimental unit, *µ* is the overall mean, *R_i_* is the *ith* experiment and *G_j_* is the *jth* genotype. The interaction between the fixed effects *G_j_* and the random effect experiment is confounded with the error *E_ij_*. The statistics software package SAS 9.3 (Copyright © 2014 SAS Institute Inc.) was used to obtain ANOVA tables, expected mean squares, and least square means for association analyses. Function PROC GLM was implemented and type 3 sums of squares were used to account for missing data. Genotypic (σ_g_^2^), and phenotypic (σ_p_^2^) variances as well as broad sense heritability (H^2^) were all calculated on an entry mean basis. Heritability on an experimental unit basis was calculated as follows:
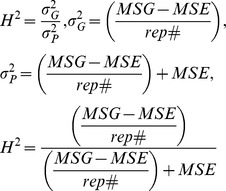



Function PROC GLM was implemented. Pearson correlations were calculated using the SAS function CORR to determine the relationships between seedling traits.

### Marker Data

Genotyping-by-sequencing (GBS) [34] was used to genotype the association mapping population with 681,257 single nucleotide polymorphism (SNP) markers across the maize genome. Imputation as described by [33] was employed. In an effort to reduce the number of non-informative markers, all monomorphic SNP markers and those with more than 20% missing data were omitted. SNP markers with a minor allele frequency less than 5% were removed, leaving 135,311 SNP markers spread across all 10 chromosomes of the maize genome to calculate population structure, kinship, and to perform GWAS.

### Association Analyses

Population structure was estimated from a reduced number of unimputed SNPs (1,665 SNP markers) using program Structure 2.3.4 [35]. Parameter settings for estimating membership of coefficients of coancestry for lines are a burn-in length of 50,000 with 50,000 iterations for each cluster (K) from 1–15, with each K being run five times. We applied an admixture model with independent allele frequencies. To pick the most probable K value, we used an ad hoc (ΔK) statistic based on the ordering rate of change of P(X|K) [36]. Software program TASSEL 4.0 [37] was used to calculate LD as well as Loiselle kinship coefficients between lines based on 135,311 SNP markers. Population structure (Q matrix) was used in association analyses to decrease the amount of type 1 errors [38]. TASSEL 4.0 was used to conduct genome wide association analyses (GWAS) using a General Linear Model (GLM) and population structure as a fixed factor with model y = Xβ+U, where y are the values measured, X is the marker value, β is a matrix of parameters to be estimated, and U uses the Q values as fixed factors. To account for multiple testing during GWAS, statistical package simpleM was implemented in R 3.0 [39]. Based on a α level of P = 0.05, the multiple testing threshold level was set to 5.3×10–7 with the equation α/n, where n equals the effective number of independent tests. Only the Ames panel was analyzed, as genomic marker data were not available for the ASI panel.

## Supporting Information

Table S1 Trait correlations between all 28 traits extracted using *ARIA*. Non-significant correlations denoted with ‘*’.
(DOCX)

Table S2 Simple statistics for all traits collected by *ARIA.*
(DOCX)
