# Supplementary material for: Analysis of Maize (Zea mays L.) Seedling Roots with the High-Throughput Image Analysis Tool ARIA (Automatic Root Image Analysis)
Source: PLoS One. 2014 Sep 24;9(9):e108255. doi: 10.1371/journal.pone.0108255 (PMC4176968; doi:10.1371/journal.pone.0108255)
Supplement: Table S1 — Trait correlations between all 28 traits extracted using ARIA. Non-significant correlations denoted with ‘*’. (DOCX) [file pone.0108255.s001.docx]

| Supplementary Table S1. Trait correlations between all 28 traits extracted using *ARIA.* Non-significant correlations denoted with ‘*’ | | | | | | | | | | | | | | | | | | | | | | | | | | | | |
| --- | --- | --- | --- | --- | --- | --- | --- | --- | --- | --- | --- | --- | --- | --- | --- | --- | --- | --- | --- | --- | --- | --- | --- | --- | --- | --- | --- | --- |
| TRL | | **SUA** | **PRL** | **SEL** | **COM** | **COP** | **CMT** | **CMM** | **CMB** | **CPT** | **CPM** | **CPB** | **MNR** | **PER** | **DEP** | **WID** | **WDR** | **MED** | **MRN** | **CVA** | **NWA** | **SOL** | **LED** | **DIA** | **VOL** | **SCS** | **SRL** | **BSH** |
| 1.000 | 0.873 | | 0.696 | 0.981 | 0.205 | -0.022* | -0.09 | 0.277 | -0.171 | -0.06 | 0.114 | -0.242 | 0.397 | 0.733 | 0.684 | 0.655 | -0.03* | 0.891 | 0.884 | 0.818 | 0.983 | .087** | -0.24 | 0.244 | 0.379 | 0.662 | -0.364 | -0.061* |
|  | 1.000 | | 0.768 | 0.872 | 0.170 | -0.10 | -0.121 | 0.254 | -.149 | -0.070 | 0.101 | -0.216 | 0.434 | 0.743 | 0.733 | 0.658 | -0.103 | 0.760 | 0.745 | 0.820 | 0.885 | -0.059* | -.232 | 0.393 | 0.465 | 0.799 | 0.500 | -0.065 |
|  |  | | 1.000 | 0.670 | 0.017* | -0.308. | -0.229 | 0.268 | -0.206 | -0.16 | 0.092 | -0.248 | 0.635 | 0.821 | 0.954 | 0.671 | -0.174 | 0.459 | 0.427 | 0.864 | 0.712 | 0.321 | -0.085 | 0.154 | 0.396 | 0.859 | -0.285 | -0.099* |
|  |  | |  | 1.000 | 0.219 | 0.006* | -0.082 | 0.276 | -0.145 | -0.057* | 0.116 | -0.219 | 0.373 | 0.721 | 0.659 | 0.654 | -0.075 | 0.925 | 0.919 | 0.811 | 0.998 | 0.042 | -0.236* | 0.255 | 0.387 | 0.681 | -0.389 | 0.065* |
|  |  | |  |  | 1.000 | 0.368 | 0.275 | 0.229 | -0.043* | 0.097 | 0.118 | -0.127 | 0.149 | 0.198 | -0.036* | 0.120 | 0.046* | 0.298 | 0.264 | 0.151 | 0.208 | 0.153 | -0.499 | 0.258 | 0.121 | 0.121 | -0.248 | 0.026* |
|  |  | |  |  |  | 1.000 | 0.282 | 0.178 | -0.011* | 0.262 | 0.330 | -0.021* | -0.13 | -0.162 | -0.363 | -0.096 | 0.146 | 0.158 | 0.166 | -0.153 | -0.019* | 0.364 | -0.345 | 0.275 | -0.007* | -0.137 | -0.249 | -0.032* |
|  |  | |  |  |  |  | 1.000 | -0.027* | 0.010* | 0.550 | 0.023 | 0.006* | -0.058* | -0.077 | -0.243 | -0.124 | -0.012* | -0.021 | 0.028* | -0.16 | -0.096 | 0.068* | -0.138 | 0.098 | -0.075 | -0.144 | 0.074* | -0.019* |
|  |  | |  |  |  |  |  | 1.000 | -0.268 | 0.017 | 0.752 | -0.278 | 0.271 | 0.306 | 0.269 | 0.235 | 0.017* | 0.262 | 0.278 | 0.287 | 0.282 | 0.051* | -0.154 | 0.176 | 0.135 | 0.259 | -0.283 | 0.009* |
|  |  | |  |  |  |  |  |  | 1.000 | -0.002* | -0.214 | 0.904 | -0.305 | -0.255 | -0.213 | -0.155 | -0.105 | -0.113 | -0.139 | -0.183 | -0.153 | -0.096 | 0.115 | -0.125 | -0.053* | -0.179 | 0.187 | -0.166 |
|  |  | |  |  |  |  |  |  |  | 1.000 | 0.047* | -0.007 | 0.114 | -0.022* | -0.169 | -0.113 | -0.036* | -0.013* | 0.044* | -0.136 | -0.066 | 0.041* | -0.093 | 0.098 | -0.057* | -0.094 | -0.104 | -0.058* |
|  |  | |  |  |  |  |  |  |  |  | 1.000 | -0.222 | 0.141 | 0.126 | 0.104 | 0.091 | 0.051* | 0.134 | 0.169 | 0.108 | 0.116 | 0.112 | -0.08 | 0.119 | 0.005* | 0.077* | -0.227 | 0.003* |
|  |  | |  |  |  |  |  |  |  |  |  | 1.000 | -0.356 | -0.304 | -0.245 | -0.196 | -0.094 | -0.185 | -0.204 | -0.244 | -0.226 | -0.101 | 0.163 | -0.123 | -0.066* | -0.204 | 0.196 | -0.202 |
|  |  | |  |  |  |  |  |  |  |  |  |  | 1.000 | 0.698 | 0.604 | 0.374 | -0.125 | 0.215 | 0.252 | 0.521 | 0.401 | -0.19 | -0.168 | 0.213 | 0.288 | 0.579 | -0.289 | -.044* |
|  |  | |  |  |  |  |  |  |  |  |  |  |  | 1.000 | 0.785 | 0.695 | -0.118 | 0.574 | 0.575 | 0.849 | 0.746 | -0.210 | -0.183 | 0.241 | 0.390 | 0.760 | -0.369 | -0.076 |
|  |  | |  |  |  |  |  |  |  |  |  |  |  |  | 1.000 | 0.582 | -0.214 | 0.428 | 0.403 | 0.811 | 0.698 | -0.317 | -0.018* | 0.049* | 0.311 | 0.771 | -0.199 | -0.110 |
|  |  | |  |  |  |  |  |  |  |  |  |  |  |  |  | 1.000 | -0.001* | 0.564 | 0.564 | 0.880 | 0.669 | -0.29 | -0.050* | 0.313 | 0.406 | 0.687 | -0.370 | -0.080 |
|  |  | |  |  |  |  |  |  |  |  |  |  |  |  |  |  | 1.000 | -0.039* | -0.031* | -0.079* | -0.021 | 0.755 | -0.043* | 0.044* | -0.016* | -0.104 | -0.072 | 0.038* |
|  |  | |  |  |  |  |  |  |  |  |  |  |  |  |  |  |  | 1.000 | 0.936 | 0.656 | 0.911 | 0.193 | -0.326 | 0.278 | 0.302 | 0.486 | -0.392 | -0.066* |
|  |  | |  |  |  |  |  |  |  |  |  |  |  |  |  |  |  |  | 1.000 | 0.632 | 0.902 | 0.210 | -0.210 | 0.309 | 0.291 | 0.462 | -0.426 | -0.058* |
|  |  | |  |  |  |  |  |  |  |  |  |  |  |  |  |  |  |  |  | 1.000 | 0.632 | -0.245 | -0.147 | 0.278 | 0.451 | 0.821 | -0.385 | -0.079 |
|  |  | |  |  |  |  |  |  |  |  |  |  |  |  |  |  |  |  |  |  | 1.000 | 0.045* | -0.236 | 0.251 | 0.386 | 0.678 | -0.388 | -0.065* |
|  |  | |  |  |  |  |  |  |  |  |  |  |  |  |  |  |  |  |  |  |  | 1.000 | -0.211 | 0.075 | -0.09 | -0.226 | -0.122 | 0.072 |
|  |  | |  |  |  |  |  |  |  |  |  |  |  |  |  |  |  |  |  |  |  |  | 1.000 | -0.364 | -0.233 | -0.229 | 0.300 | -0.004* |
|  |  | |  |  |  |  |  |  |  |  |  |  |  |  |  |  |  |  |  |  |  |  |  | 1.000 | 0.450 | 0.587 | -0.808 | 0.104 |
|  |  | |  |  |  |  |  |  |  |  |  |  |  |  |  |  |  |  |  |  |  |  |  |  | 1.000 | 0.499 | -0.407 | -.005* |
|  |  | |  |  |  |  |  |  |  |  |  |  |  |  |  |  |  |  |  |  |  |  |  |  |  | 1.000 | -0.594 | -0.068* |
|  |  | |  |  |  |  |  |  |  |  |  |  |  |  |  |  |  |  |  |  |  |  |  |  |  |  | 1.000 | -0.032* |
|  |  | |  |  |  |  |  |  |  |  |  |  |  |  |  |  |  |  |  |  |  |  |  |  |  |  |  | 1.000 |
