# Supplementary material for: Analysis of Maize (Zea mays L.) Seedling Roots with the High-Throughput Image Analysis Tool ARIA (Automatic Root Image Analysis)
Source: PLoS One. 2014 Sep 24;9(9):e108255. doi: 10.1371/journal.pone.0108255 (PMC4176968; doi:10.1371/journal.pone.0108255)
Supplement: Table S2 — Simple statistics for all traits collected by ARIA. (DOCX) [file pone.0108255.s002.docx]

| Supplementary Table S2. Simple statistics for all traits collected by *ARIA* | | | | | |
| --- | --- | --- | --- | --- | --- |
| Trait | **Mean** | **Std. Dev** | **Minimum** | **Maximum** | **H^2^** |
| TRL | 190.94 cm | 98.73 | 16.39 cm | 536.33 cm | 0.42 |
| SUA | 10.22 cm^2^ | 4.32 | 1.16 cm^2^ | 25.04 cm^2^ | 0.42 |
| PRL | 28.45 cm | 8.35 | 4.09 cm | 47.06 cm | 0.28 |
| SEL | 149.32 cm | 92.80 | 0.16 cm | 490.59 cm | 0.42 |
| COM | 0.39 | 0.11 | 0.10 cm | 0.78 | 0.10 |
| COP | 0.43 | 0.07 | 0.18 | 0.74 | 0.44 |
| CMT | 0.18 | 0.04 | 0 | 0.32 | 0.11 |
| CMM | 0.49 | 0.06 | 0 | 0.62 | 0.13 |
| CMB | 0.79 | 0.08 | 0 | 0.99 | 0.04 |
| CPT | 0.19 | 0.02 | 0 | 0.30 | 0.10 |
| CPM | 0.48 | 0.05 | 0 | 0.62 | 0.12 |
| CPB | 0.82 | 0.08 | 0 | 0.99 | 0.04 |
| MNR | 80.80 | 33.94 | 4.00 | 196 | 0.39 |
| PER | 143.38 cm | 54.06 cm | 9.77 cm | 307.07 cm | 0.31 |
| DEP | 24.17 | 6.54 | 3.56 | 34.88 | 0.25 |
| WID | 5.23 | 1.64 | 0.81 | 10.50 | 0.49 |
| WDR | 0.25 | 0.42 | 0.08 | 13.01 | 0.27 |
| MED | 5.12 | 2.61 | 1.00 | 16.00 | 0.45 |
| TNR | 11.05 | 4.94 | 1.00 | 26.67 | 0.49 |
| CVA | 87.79 | 43.36 | 1.24 | 218.90 | 0.30 |
| NWA | 1.09 | 0.61 | 0.03 | 3.26 | 0.39 |
| SOL | 0.01 | 0.01 | 0.01 | 0.19 | 0.04 |
| BSH | 100.89 | 1582 | 1.00 | 327.69 | 0.12 |
| LED | 0.76 | 0.31 | 0.02 | 3.13 | 0.19 |
| DIA | 0.12 | 0.03 | 0.05 | 0.35 | 0.32 |
| VOL | 62.22 | 86.46 | 0.05 | 796.35 | 0.08 |
| SRL | 0.59 | 0.40 | 0.05 | 2.54 | 0.21 |
